# Knee Joint Vibration Signal Analysis with Matching Pursuit Decomposition and Dynamic Weighted Classifier Fusion

**DOI:** 10.1155/2013/904267

**Published:** 2013-03-12

**Authors:** Suxian Cai, Shanshan Yang, Fang Zheng, Meng Lu, Yunfeng Wu, Sridhar Krishnan

**Affiliations:** ^1^School of Information Science and Technology, Xiamen University, 422 Si Ming South Road, Xiamen, Fujian 361005, China; ^2^Department of Electrical and Computer Engineering, Ryerson University, 350 Victoria Street, Toronto, ON, Canada M5B 2K3

## Abstract

Analysis of knee joint vibration (VAG) signals can provide quantitative indices for detection of knee joint pathology at an early stage. In addition to the statistical features developed in the related previous studies, we extracted two separable features, that is, the number of atoms derived from the wavelet matching pursuit decomposition and the number of significant signal turns detected with the fixed threshold in the time domain. To perform a better classification over the data set of 89 VAG signals, we applied a novel classifier fusion system based on the dynamic weighted fusion (DWF) method to ameliorate the classification performance. For comparison, a single leastsquares support vector machine (LS-SVM) and the Bagging ensemble were used for the classification task as well. The results in terms of overall accuracy in percentage and area under the receiver operating characteristic curve obtained with the DWF-based classifier fusion method reached 88.76% and 0.9515, respectively, which demonstrated the effectiveness and superiority of the DWF method with two distinct features for the VAG signal analysis.

## 1. Introduction

 The knee is the largest and most complex joint in the human body [1]. A pair of knees support nearly the entire weight of the human body and help the body perform different locomotion functions. Knee osteoarthritis is a common form of rheumatic disorder caused by the degeneration or damage of articular cartilage in the knee joint. Detection of knee joint pathology at an early stage can help clinicians apply appropriate therapeutical or surgical procedures to retard the degenerative process in the affected knee joint [2, 3].

Arthroscopy is usually performed as a semi-invasive surgical procedure for knee joint disorder detection [1]. Physicians can inspect the interior of a joint with an arthroscope fiber that is inserted into the knee through a small incision. Although arthroscopy is often used as the gold standard for relatively low-risk assessment of joint surfaces [4], it cannot be applied to patients whose knees are in a highly degenerated state due to arthritis, ligamentous instability, meniscectomy, or patellectomy. Furthermore, arthroscopy is not suited for routine examinations of the articular cartilage because the same incision may not undergo repeated arthroscope fiber invasions due to bacterial infection.

Magnetic resonance imaging can assist in the characterization of *in vivo* orthopaedic condition of articular cartilage and is also a popular noninvasive method for the assessment of knee joint degeneration [5]. The weakness of the magnetic resonance imaging is that such a technique is only able to display anatomical morphology [6]. The imaging protocol cannot support functional condition detection of the knee joint during leg movement, because the subject has to lay down throughout the magnetic resonance scanning procedure.

Vibration arthrometry is an alternative technology for noninvasive detection of knee pathologies [4, 7]. The knee joint vibration arthrographic (VAG) signal can be recorded by accelerometer or electrostethoscope sensors attached on the surface of the knee cap [8–10]. For the healthy adults, their articular surfaces in the knee are smooth and slippery without any cartilage friction or collision. However, the vibrations generated due to the friction between the degenerative articular cartilages are expected to present anomalous patterns in the amplitude and frequency scales [2]. The vibration arthrometry not only provides a clinical option of the noninvasive and low-cost routine detection for knee joint disorders but also supports the functional study on the knee joint during leg movements [1].

Computer-aided analysis of knee joint VAG signals is very useful for screening and monitoring of articular cartilage disorders at an early stage [11–13]. Based on the noninvasive detection results, the computational algorithms may effectively help the medical experts make an accurate decision, so that the frequency of the diagnostic open surgery with arthroscope can be reduced [8, 14–16]. Adaptive filtering techniques based on the least-mean-square (LMS) and recursive least-squares lattice (RLSL) algorithms were used to remove muscle contraction interference present in VAG signals [17]. Tavathia et al. [18] and Moussavi et al. [19] proposed different linear prediction models and adaptive segmentation methods for parameterization and screening of VAG signals. Jiang et al. [20] extended the application of vibration arthrometry to artificial knee joints *in vitro* and analyzed the VAG signal with the root-mean-squared (RMS) value and the parameters of an autoregressive (AR) model. Matching pursuit (MP) time-frequency distributions (TFDs) of VAG signals were studied by Krishnan et al. [9], and a modified local discriminant bases algorithm was proposed by Umapathy and Krishnan [14] to distinguish the abnormal VAG signals from the normal ones. In order to simplify the procedures of signal processing and decision making, Rangayyan and Wu [11, 12] proposed the statistical parameters including form factors, skewness, kurtosis, probability density function entropy, variance of mean-squared values, and turns count with adaptive threshold, for the screening of VAG signals based on the radial-basis function network (RBFN). Mu et al. [21] used the linear and nonlinear strict 2-surface proximal classifiers to test a subset of the aforementioned statistical parameters. In this paper, the number of atoms derived from the wavelet MP decomposition and the turns count detected with the fixed threshold in the waveform variability analysis are extracted as features, and a classifier fusion system based on the dynamic weighted fusion (DWF) method is proposed for the classification of the VAG signals.

## 2. Data Set

 The VAG data were collected by the research group of Rangayyan, University of Calgary, Canada [9]. The experimental protocol was approved by the Conjoint Health Research Ethics Board of the University of Calgary. Each subject was requested to sit on a rigid table in a relaxed position with the leg being tested freely and suspended in midair. The knee joint vibration was measured by placing a miniature accelerometer at the middle position of the patella [8]. Minute electrical charges were generated by the accelerometer based on the acceleration and deceleration of the knee movement when the subject swung the leg over an approximate angle range of 135° to 0° and back to 135° in the duration *T* = 4 s. Each VAG signal was conditioned by an isolation preamplifiers to prevent the aliasing effects. The signal was then amplified and digitized with a data acquisition board and the National Instruments LabVIEW software at the sampling rate *f*_*s*_ = 2 kHz with 12-bit resolution per sample. Auscultation of the knee joint using a stethoscope was also performed, and a qualitative description of the sound intensity and type was recorded.

**Figure 1:**
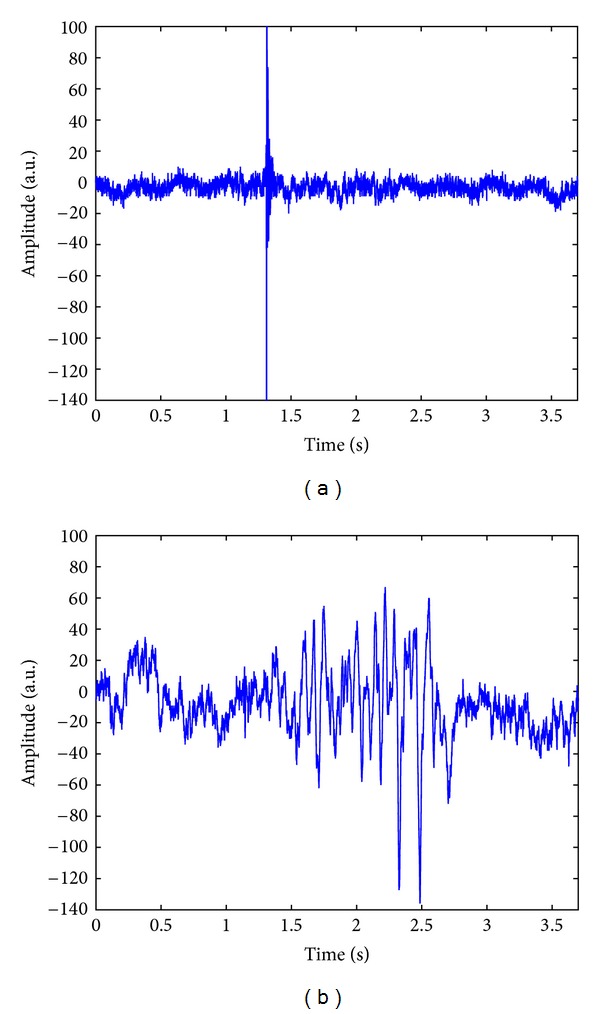
Raw knee joint vibration signals of (a) a healthy subject and (b) a patient with knee joint pathology. a.u.: uncalibrated acceleration units.

In the present study, we used a total of 89 VAG signals (recorded from 51 healthy volunteers and 38 subjects with knee joint pathologies), the same as investigated in a few previous related studies [11, 12, 14]. The normal signals were recorded from the healthy subjects identified by physical examinations. The abnormal signals, an example of which is shown in Figure 1(b), were collected from symptomatic patients scheduled to undergo arthroscopic examinations independent of the VAG studies. The knee joint disorders were associated with chondromalacia of different grades, meniscal tear, tibial chondromalacia, and anterior cruciate ligament injuries, which were confirmed in arthroscopy. Compared with the normal VAG signal displayed in Figure 1(a), we can observe that the abnormal signal exhibits a higher degree of variability in the time domain, as illustrated in Figure 1(b).

## 3. Feature Description

### 3.1. Number of Wavelet Matching Pursuit Decomposition Atoms

VAG signals are nonstationary in nature, that is, such signals exhibit time-varying spectral characteristics, and they cannot be accurately represented by common signal processing techniques such as Fourier transform and AR modeling [9]. It is therefore better to use a joint time-frequency approach for VAG analysis. For time-frequency representation of a signal, the methodology of MP introduced by Mallat and Zhang [22] is able to decompose the signal using basis functions with good time-frequency properties which are referred to as atoms.

The MP method is a so-called “greedy” algorithm that successively approximates a signal *x*(*t*) of *N* samples with orthogonal projections onto elements from a waveform dictionary *𝒟* = {*d*_*r*_(*t*)}_*r*∈Γ_ of *P* vectors, in which $||d_{r}||=\sqrt{\left[\int d_{r}^{2}(t)dt\right]}=1$. Gabor function, local cosine trees, and wavelet packets are often applied to build up dictionaries for MP applications. In this investigation, we implemented the Daubechies wavelet MP decomposition [23], because the Daubechies wavelets are a family of orthogonal wavelets that have a support of minimum size for a given number of vanishing moments [24], and such wavelets can be used for signal decomposition with excellent time and scale properties [25]. The projection of VAG signal *x*(*t*) using the dictionary of wavelet packet bases, *d*_*r*_*m*__(*t*), calculated with a Daubechies 8 (db8) filter can be formulated as

(1) $$\begin{array}{c} x\left(t\right)=\sum_{m=0}^{M-1}a_{m}d_{r_{m}}\left(t\right), \end{array}$$

where *a*_*m*_ are the expansion coefficients and *M* denotes the iterations of decomposition. And the wavelet MP decomposition can be implemented as follows. In the beginning, *x*(*t*) is projected on a vector *d*_*r*_0__(*t*) ∈ *𝒟*, and the residue *R* ^1^ *x*(*t*) is computed, that is,

(2) $$\begin{array}{c} x\left(t\right)=\langle x,d_{r_{0}}\rangle d_{r_{0}}\left(t\right)+R^{1}x\left(t\right), \end{array}$$

where 〈*x*, *d*_*r*_0__〉 denotes the inner product (projection). Since the first atom *d*_*r*_0__(*t*) is orthogonal to *R* ^1^ *x*(*t*), we have

(3) $$\begin{array}{c} {||x||}^{2}={\left|\langle x,d_{r_{0}}\rangle \right|}^{2}+{||R^{1}x||}^{2}. \end{array}$$

In order to minimize ||*R* ^1^ *x*||, *r*_0_ ∈ Γ is chosen such that |〈*x*, *d*_*r*_0__〉| is maximum, that is,

(4) $$\begin{array}{c} \left|\langle x,d_{r_{0}}\rangle \right|\geq \underset{r\in \Gamma }{\sup}\left|\langle x,d_{r}\rangle \right|. \end{array}$$

The MP iterates this procedure by subdecomposing the residue. And the VAG signal *x*(*t*) after *M* iterations of decomposition is then expressed as

(5) $$\begin{array}{c} x\left(t\right)=\sum_{m=1}^{M-1}\langle R^{m}x,d_{r_{m}}\rangle d_{r_{m}}\left(t\right)+R^{M}x\left(t\right), \end{array}$$

where |〈*x*, *d*_*r*_*m*__〉| ≥ sup⁡_*r*∈Γ_⁡|〈*x*, *d*_*r*_〉| and *R* ^0^ *x*(*t*) ≡ *x*(*t*). Since the residue term *R* ^*M*^ *x*(*t*) can be regarded as noise after sufficient iterations, one common approach to stop the iterative process depends on the convergence of residual energy, ||*R* ^*m*^ *x*||^2^. In the previous related studies [9, 26], Krishnan et al. used the decay parameter defined by Mallat and Zhang [22] to end the MP decomposition based on the Gabor function dictionary. Although the decay parameter as an iterative indicator is well devised in theory, for a wavelet packet dictionary that contains *P* = *N*  log⁡_2_ *N* vectors, each MP iteration then requires *O*(*N* log⁡_2_ *N*) operations. For the VAG data analyzed, each signal consists of *N* = *f*_*s*_ × *T* = 8000 samples, such that the wavelet MP decomposition is computationally expensive.

In the present study, we propose using a signal-to-noise ratio (SNR) as the alternative indicator to determine the iterations of the wavelet MP decomposition. According to the VAG signals in Figure 1, it can be inferred that, with a given SNR, the number of the wavelet MP decomposition iterations for a normal signal will be fewer than that for an abnormal signal, because the abnormal signal is much more noisy and also contaminated by a larger amount of artifacts such as muscle contraction interference. Thus, the number of MP iterations can be considered as a potential feature for classification applications. On the other hand, the MP decomposition with many great iterations can provide an excellent value of SNR and is also suited for denoising applications [26], but such an implementation is time consuming as mentioned above. It is therefore necessary to search for an appropriate value of SNR that makes a tradeoff between efficiency and effectiveness of the wavelet MP decomposition. After testing the SNR with different values, we found that the SNR of 15 dB could be an excellent indicator to determine the wavelet MP iterations. The *P* value (0.0002) of the number of atoms (Natom) obtained with the Student's *t*-test [27] indicated that the Natom values (numerically equal to the number of decomposition iterations *M*) were significantly different between the normal and abnormal VAG signals. Such results also confirm our assumption mentioned above.

**Figure 2:**
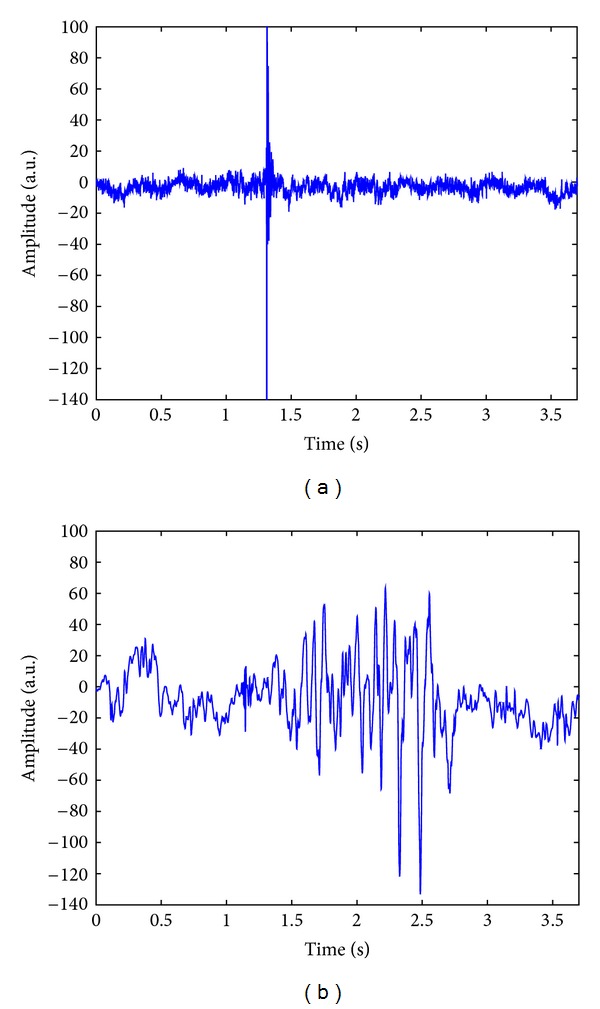
Reconstructed VAG signals with the wavelet matching pursuit atoms with regards to the original records in Figure 1: (a) of a healthy subject; (b) of a patient with knee joint pathology. a.u.: uncalibrated acceleration units.

From Figure 2, we can observe that the much noise has been reduced in the normal and abnormal VAG signals, reconstructed with 145 and 784 MP atoms, respectively. The results of noise removal in the VAG signals, as depicted in Figure 2, are even better than those in the previous study using the Gabor function dictionary and the energy decay parameter [26].

### 3.2. Turns Count with the Fixed Threshold

Besides the time-frequency MP decomposition, the waveform variability analysis in the time domain may be useful for classification as well. According to Rangayyan [28], a signal sample can be identified as a “turn” if it satisfies the following two conditions at the same time: (1) it represents a change in direction in the signal, that is, an alteration in the sign of the derivative (from positive to negative or vice versa); (2) the difference between its amplitude and that of the preceding sample is over a certain threshold. Willison [29] used the turns count method to analyze the electromyographic signal. The experiments showed that the electromyographic signal recorded from a patient with myopathy usually possesses more turns than the signal of a healthy subject at a comparable level of volitional effort. In the present work, we first normalized each VAG signal in the amplitude range from zero to unity, the same as in the recent studies [11, 12]. In each signal, the amplitude of all samples was amplified with the same scale, so that the variability information of the signal can be preserved. Before applying the turns count method in the signal, we implemented a filtering procedure using a 10th-order lowpass Butterworth filter (cutoff frequency: 50 Hz) with unit gain at direct current (DC) [23]. This lowpass Butterworth filter causes a delay of 100 samples (or 0.05 s), which was calibrated after the filtering procedure in our experiments. The reason that we used the lowpass Butterworth filter instead of the signal reconstructed with the MP atoms, as shown in Figure 2, is that the MP method is unable to eliminate the artifacts such as the interference caused by muscle contractions or 50 or 60 Hz power-supply lines.

In the past work of Rangayyan and Wu [12], the threshold to determine the significance of a turn was adaptively set to be 0.5*σ*_v_, where *σ*_v_ denotes the standard deviation of the VAG signal analyzed. Although the turns detected with the adaptive threshold provide good discriminant information for classification of  VAG signals, the number of turns counted from a normal signal is larger than that of an abnormal one, because the standard deviation of the normal VAG signal is usually smaller than that of the abnormal signal [12]. Such a result, however, somewhat deviates from our expectation that the turns associated with an abnormal VAG signal would be larger in number due to a higher degree of variability. In the present study, we fixed the amplitude threshold at 0.2 to compute the turns over the normalized and filtered VAG signals.

**Figure 3:**
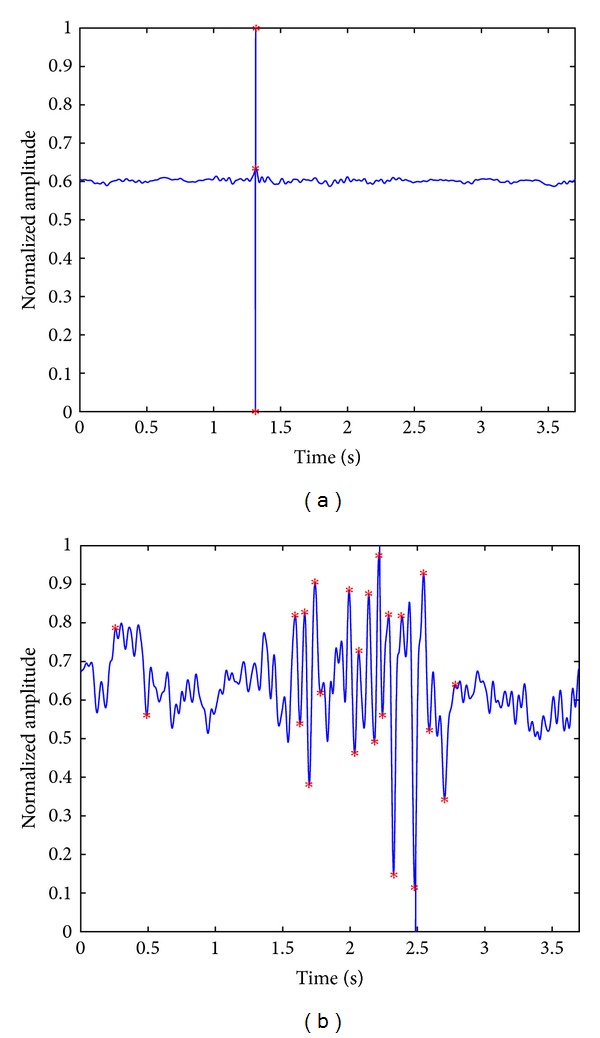
Illustration of significant turns in the filtered (a) normal and (b) abnormal VAG signals, in which the significant turns detected have been marked with asterisks. The delay of 0.05 s caused by the lowpass Butterworth filter has been calibrated.

Figure 3 shows the results of the turns count with the fixed threshold (TCFT) method for the VAG signals in Figure 1, which were normalized and processed with the Butterworth filter. We can observe that more significant turns have been identified in the abnormal signal, as marked in Figure 3(b), in comparison with the normal signal shown in Figure 3(a). In addition, the *P* value of the TCFT obtained with the Student's *t*-test is 0.0013 (significance level: *P* < 0.01), which indicates a significant difference between the normal and abnormal signals.

### 3.3. Statistical Features

In addition to two aforementioned features, we also considered the other five features extracted from the same VAG data set in our previous work [11–13, 30]. These features included the form factors computed for the first half (FF1) and the second half (FF2) of each VAG signal [11]; the variance of the mean-squared value (VMS) of the each signal [12]; the mean value (*μ*) of the Parzen-window probability density function of each signal [13]; the fractal dimension (FD) estimated by the power spectral analysis [30]. Total seven features were combined in the vector form for the following pattern analysis task.

## 4. Classification

 To perform the signal classifications, we applied a single least-squares support vector machine (LS-SVM) and the ensembles of several component LS-SVM classifiers, the details of which are presented as follows.

### 4.1. Least-Squares Support Vector Machine

The support vector machine (SVM) proposed by Cortes and Vapnik [31] is a type of universal approximator, the learning of which follows the structural risk minimization criterion [32]. To optimize the SVM model parameters, a subset of the representative training data is selected to be the support vectors, which are considered to be the most informative for the classification task. By choosing the nonlinear inner-product kernels in the network, the SVM is able to perform the same function as the polynomial learning machine, radial basis function network, or multilayer perceptron with a single hidden layer [33, 34]. The LS-SVM was proposed by Suykens et al. [35] as a reformulation to the standard SVM, with an improvement of the moderate complexity. The learning of the LS-SVM is implemented by minimizing a regularized least-squares cost function with equality constraints, under the Kuhn-Tucker condition [36]. Recently, the LS-SVM has also been widely used in a number of biomedical applications [37–39].

To determine the kernel function suited for the VAG signal classification, we implemented the LS-SVM using the linear, polynomial, sigmoid, and Gaussian kernels, one by one specified by different model parameters, and then evaluated each LS-SVM with the leave-one-out (LOO) method. As a type of cross-validation approach, the LOO partition procedure repeatedly used each signal once for the validation and the remaining signals for training [34]. By checking the accuracy and the optimal separating hyperplane provided by each LS-SVM, we chose the polynomial kernel function, the degree and intercept parameters of which equal to 2 and 1, respectively, and set the regularization parameter of the LS-SVM to be 5.

### 4.2. Ensemble of LS-SVMs with the Bagging Algorithm

As an emerging machine learning methodology based on the “divide and conquer” principle [33], ensemble of classifiers was widely used in the literature [40–44], with the aim to achieve a better performance versus a single classifier. By combing a finite number of component neural networks (CNNs) with a well-devised combination rule [41–43] or fusion strategy [44–46], a neural network ensemble is expected to provide an informative overall decision that is supposedly superior to that attained by any one of the CNNs acting solely [33].

The most popular ensemble algorithms are AdaBoost [47] and Bagging [48]. The AdaBoost works by repeatedly training a given type of weak-learning machine from different distributed training data sets and then combining their outputs. The distribution of training data for the current CNN is boosted depending on the performance of previous CNNs, that is, the training data that are incorrectly predicted by previous CNNs will be chosen with priority to train the current CNN. In spite of the effectiveness, the AdaBoost is very sensitive to outliers and sometimes results in overfitting [49]. On the other hand, the Bagging algorithm introduces the bootstrap approach [50] into the training data resampling procedure [48] and aggregates the CNNs with the simple average strategy [33]. In the bootstrap procedure, each data sample was selected separately at random from the original data set such that a particular data sample could appear multiple times in a bootstrap-generated data set. The bias of the Bagging ensemble would converge by averaging, while the variance falls much smaller than that of each CNN.

Since the LS-SVM is not a weak-learning machine [35], we used the Bagging algorithm rather than the AdaBoost for the ensemble of 5 component LS-SVMs (CSVMs) which were labeled from CSVM1 to CSVM5 in numerical sequence. The CSVMs combined with the Bagging algorithm were trained by different bootstrap-generated data sets. The number of signals in each bootstrap-generated data set was of equal size to the original VAG data set. The testing data set for each CSVM was the same as the original VAG data set. Because the training data for each CSVM were generated using the bootstrap approach, it is not necessary to apply the LOO method to the ensemble system any more.

### 4.3. Ensemble of LS-SVMs with the Dynamic Weighted Fusion Rule

According to the linear combination rule of the Bagging, the CSVMs are simply averaged in the ensemble, so that the effectiveness of the ensemble would be affected by some of the CSVMs with poor performance, because the simple average strategy treats all the CSVMs equally. With the aim to utilize the diverse knowledge generated by the CSVMs, we applied a dynamic weighted fusion (DWF) rule to adaptively combine the CSVMs in the classifier fusion system [51].

Suppose that a total of *K* CSVMs are linearly combined in the classifier fusion system. The local decision generated by the *k*th CSVM is denoted as *g*_*k*_(**f** ^*n*^), with regard to the feature vector of the *n*th VAG signal, **f** ^*n*^. The classifier fusion system then provides the overall classification decision, *g*_DWF_(**f** ^*n*^), by linearly combining the CSVMs with the weights *w*_*k*_(**f** ^*n*^) that are varied from one signal to another. Thus, the DWF-based ensemble output can be formulated as

(6) $$\begin{array}{c} g_{\text{DWF}}\left(f^{n}\right)=\sum_{k=1}^{K}w_{k}\left(f^{n}\right)g_{k}\left(f^{n}\right). \end{array}$$

The nonnegative and normalization constraints on the fusion weights, as widely accepted in the literature [41, 43, 45], can be written as

(7) $$\begin{array}{c} \sum_{k=1}^{K}w_{k}\left(f^{n}\right)=1,\;w_{k}\left(f^{n}\right)\geq 0. \end{array}$$

The task of the DWF is to determine the fusion weights that help the ensemble system provide an overall classification decision with higher accuracy. To achieve this goal, let us study the error term of the CSVMs and the DWF-based ensemble. Concerning the *k*th CSVM, the squared error that characterizes the difference between the local decision and the desired class label, *l*(**f** ^*n*^), in relation to the *n*th VAG signal is

(8) $$\begin{array}{c} e_{k}^{2}\left(f^{n}\right)={\left[l(f^{n})-g_{k}(f^{n})\right]}^{2}. \end{array}$$

Then, the squared error of the ensemble, *e*_DWF_^2^(**f** ^*n*^), is estimated in an analogous manner to that of each CSVM. Consider that the fusion weights are normalized, as presented in (7), the class label *l*(**f** ^*n*^) can be split by multiplying the fusion weights, so that *e*_DWF_^2^(**f** ^*n*^) is derived as follows:

(9) eDWF2(fn)=[l(fn)−∑k=1Kwk(fn)gk(fn)]2=[∑k=1Kwk(fn)l(fn)−∑k=1Kwk(fn)gk(fn)]2={∑k=1Kwk(fn)[l(fn)−gk(fn)]} ×{∑j=1Kwj(fn)[l(fn)−gj(fn)]}=∑k=1 K∑j=1Kwk(fn)wj(fn)[l(fn)−gk(fn)]   ×[l(fn)−gj(fn)]=∑k=1 K∑j=1Kwk(fn)wj(fn)ek(fn)ej(fn),

where *e*_*k*_(**f** ^*n*^) = [*l*(**f** ^*n*^) − *g*_*k*_(**f** ^*n*^)] represents the instantaneous error of the *k*th CSVM.

Considering (7) and (9), the minimization of the squared error of the ensemble is equivalent to the constrained quadratic programming (CQP) problem specified as follows:

(10) minimize eDWF2(fn)=∑k=1 K∑j=1Kwk(fn)wj(fn)ek(fn)ej(fn),subject to ∑k=1Kwk(fn)=1, wk(fn)≥0.

In order to solve the CQP problem presented in (10), we applied the Lagrange multiplier method [36] and defined the cost function as

(11) C(w1(fn),…,wK(fn),λ(fn))  =∑k=1 K∑j=1Kwk(fn)wj(fn)ek(fn)ej(fn)   −λ(fn)[∑k=1Kwk(fn)−1],

where the nonnegative coefficient *λ*(**f** ^*n*^) represents the Lagrange multiplier, which varies from one signal to another.

According to the weak Lagrangian principle [36], the optimum solution to the CQP problem, {w*(**f** ^*n*^), λ*(**f** ^*n*^)}, is the stationary point of the cost function presented in (11) and satisfies the following unique equations:

(12) ∂C(w1(fn),…,wK(fn),λ(fn))∂wk(fn)  =2∑j=1Kwj(fn)ek(fn)ej(fn)−λ(fn)=0,∂C(w1(fn),…,wK(fn),λ(fn))∂λ(fi)  =∑k=1Kwk(fn)−1=0.

The optimal weights *w*_*k*_(**f** ^*n*^) of the DWF that minimize the squared error of the ensemble system can be obtained by solving (12), that is,

(13) $$\begin{array}{c} w_{k}^{*}\left(f^{i}\right)=\frac{\sum_{j=1}^{K}e_{k}^{-1}\left(f^{n}\right)e_{j}^{-1}\left(f^{n}\right)}{\sum_{i=1}^{K}\sum_{j=1}^{K}e_{i}^{-1}\left(f^{n}\right)e_{j}^{-1}\left(f^{n}\right)},\;k=1,\ldots ,K. \end{array}$$

Because the error term of the CSVM can be estimated when the *n*th VAG class label *l*(**f** ^*n*^) is given and the CSVM model parameters are specified, the optimal fusion weights can be directly computed according to (13).

Now let us divert our attention to the DWF-based ensemble error term. Considering (9) and (13), we have

(14) eDWF2(fn)=∑k=1 K∑j=1Kwk(fn)wj(fn)ek(fn)ej(fn)     =1∑i=1K∑j=1Kei−1(fn)ej−1(fn).

It is clear that both of *e*_*k*_^2^(**f** ^*n*^) and *e*_DWF_^2^(**f** ^*n*^) are nonnegative, that is, *e*_*k*_^2^(**f** ^*n*^) ≥ 0 and ∑_*i*=1_^*K*^∑_*j*=1_^*K*^ *e*_*i*_^−1^(**f** ^*n*^)*e*_*j*_^−1^(**f** ^*n*^) ≥ 0. To compare with the squared error of the CSVM, we may employ the division operator such that

(15) $$\begin{array}{c} \frac{e_{k}^{2}\left(f^{n}\right)}{e_{\text{DWF}}^{2}\left(f^{n}\right)}=1+\sum_{\begin{array}{c} i=1\\ i\neq k\; \end{array}}^{K}\sum_{\begin{array}{c} j=1\\ j\neq k \end{array}}^{K}\frac{e_{k}^{2}\left(f^{n}\right)}{e_{i}\left(f^{n}\right)e_{j}\left(f^{n}\right)}\geq 1. \end{array}$$

Therefore, the optimal fusion weights derived in (15) guarantee that the DWF-based ensemble system more or less likely outperforms a single CSVM.

For a fair performance comparison, the CSVMs combined by the DWF-based ensemble were with the same bootstrap-generated training data and model parameters as those CSVMs combined by the Bagging algorithm. The testing data for the CSVMs were the entire data set of the VAG signals.

**Figure 4:**
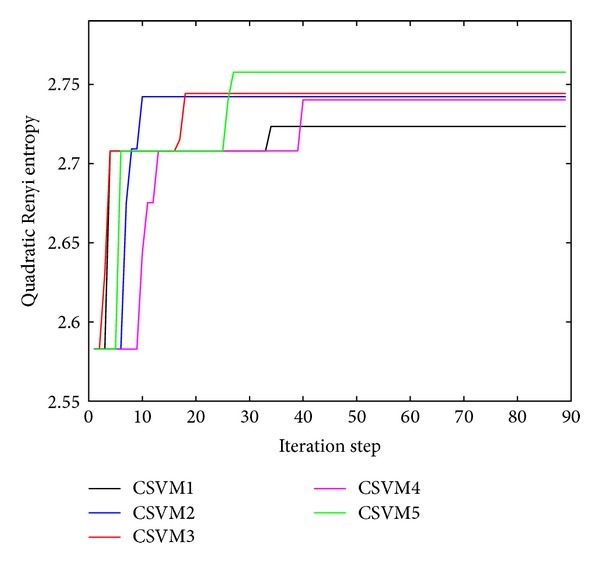
Quadratic Renyi entropy values with respect to the iteration steps. The training data of the fixed size 15 for each component LS-SVM was randomly generated and then selected according to the quadratic Renyi entropy maximization criterion.

 In addition, we also selected some subsets of fixed size 15 to train the CSVMs, and would like to evaluate the generalization capability of the proposed DWF-based ensemble in the case of small-size training data. The subset for each CSVM was actively selected according to the quadratic Renyi entropy maximization criterion [52] as follows. In the experiments, we first randomly partitioned the entire VAG data set into two subsets: the working subset containing 15 VAG signals and the candidate subset containing the remaining 74 signals. In each iteration step, we randomly selected one VAG signal **f** ^*c*∗^ from the candidate subset to replace an arbitrary signal **f** ^*w*∗^ in the working subset. If the quadratic Renyi entropy of the new working subset increases, then the working signal **f** ^*w*∗^ should be replaced by the candidate signal **f** ^*c*∗^; otherwise, **f** ^*w*∗^ remains in the working subset, and the candidate signal **f** ^*c*∗^ should be rejected and returned to the candidate subset [35]. After a few iteration steps, the quadratic Renyi entropy would become stable when reaching the maximum value, then the active selection of the fixed-size subset is terminated. The subsets for the 5 CSVMs were selected independently, and the corresponding quadratic Renyi entropy values with respect to the iteration steps are showed in Figure 4. After the model parameters of the CSVMs were optimized by the corresponding subsets of the fixed size, the original VAG data were input to the CSVMs and the DWF-based ensemble for testing and comparison purpose.

## 5. Classification Results

**Figure 5:**
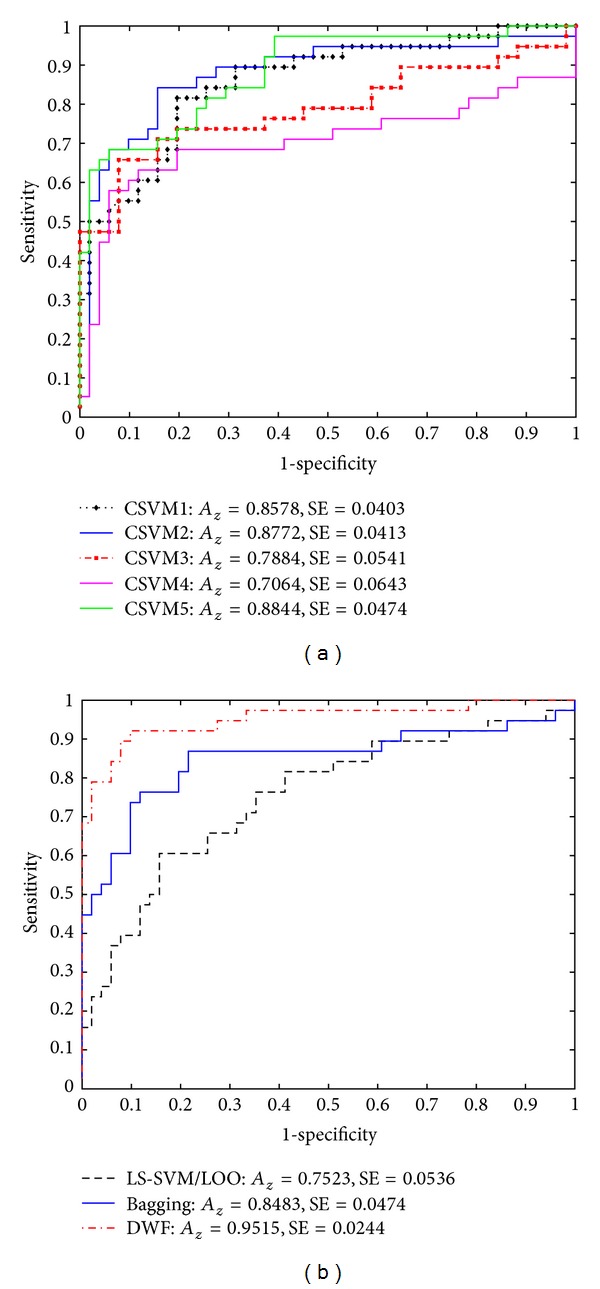
ROC curves provided by (a) the five component LS-SVM classifiers in the ensemble; (b) the LS-SVM evaluated by the leave-one-out method, the Bagging, and the dynamic weighted fusion (DWF) method. The ROC results were obtained with the testing data.

 The classification accurate rate in percentage of the single LS-SVM with the LOO method (LS-SVM/LOO) was 87.64%, which was better than the result (accuracy: 61.8%) obtained by the Fisher's linear discriminant analysis using the same feature set. In addition, the receiver operating characteristic (ROC) curve technique was implemented to test the overall diagnostic performance for all classification methods. According to Table 1, the area under the ROC curve (*A*_*z*_) obtained with the LS-SVM/LOO was 0.7523 with a standard error (SE) of 0.0536, as illustrated in Figure 5.

In the ensemble experiments, the highest accuracy provided the CSVMs was 83.15%, whereas the lowest one was only 76.41%. Despite that the *A*_*z*_ value of the CSVM4 was slightly smaller than that of the LS-SVM/LOO, any of the other four CSVMs outperformed the LS-SVM/LOO with higher *A*_*z*_ values under the ROC curves (see Figure 5).

Referring to Table 1 and Figure 5, the superiority of the DWF-based ensemble in the diagnostic performance was prominent. The DWF-based ensemble provided the highest overall accuracy of 88.76%, the best *A*_*z*_ value of 0.9515, and the lowest SE of 0.0244. It can be observed from Figure 5 that the ROC curve obtained with the DWF-based ensemble was consistently over either of that provided by the Bagging or the single LS-SVM/LOO classifier. In comparison with the single LS-SVM/LOO classifier, the Bagging ensemble can improve the diagnostic ROC curve, but its accurate rate was even 8.99% lower than that of the LS-SVM/LOO classifier.

**Figure 6:**
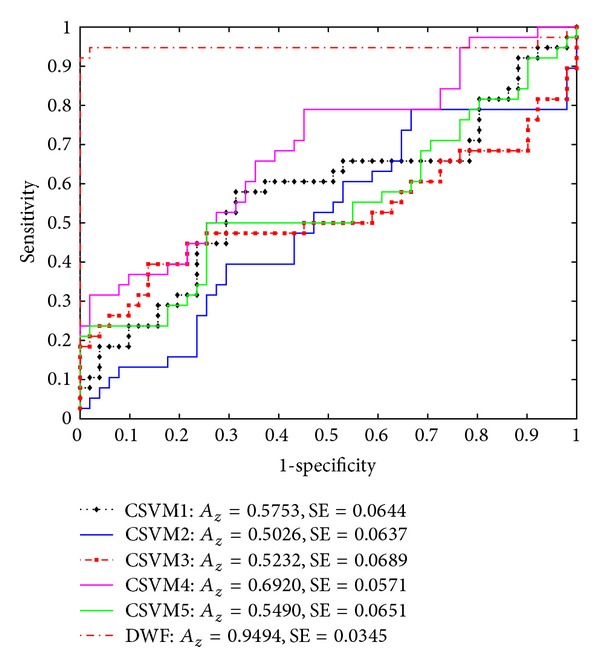
ROC curves of the testing data provided by the five component LS-SVM classifiers and the dynamic weighted fusion (DWF) ensemble. The model parameters of the component LS-SVM were optimized with the training data of fixed size 15 selected according to the quadratic Renyi entropy maximization criterion. The ROC results were obtained with the testing data.

Regarding the experiments with small-size training data input, the CSVMs optimized by the training data of fixed size 15 provided relatively poor diagnostic results on the testing data (the entire VAG data set), as depicted in Figure 6. The highest *A*_*z*_ value produced by CSVM4 was only 0.692 (SE: 0.0571), and the worst *A*_*z*_ value produced by CSVM2 was 0.5026 (SE: 0.0637), the latter of which was just slightly better than a wild guess. However, the DWF-based ensemble was not affected by its CSVMs which had difficulty in the case of lacking enough training data. The *A*_*z*_ value under the ROC curve provided by the DWF-based ensemble still reached as high as 0.9494, with the SE value of 0.0345. Such results indicated that the DWF-based ensemble had a better generalization capability when coping with the training data of smaller size.

## 6. Discussion

The Natom and TCFT features derived in the present study indicated the significance of difference (*P* < 0.01) between the normal and abnormal VAG signals, and helped the classifiers improve their diagnostic performance. In the previous work [12], the signal turns were adaptively determined by the threshold that was a half of the standard deviation of the VAG signal analyzed. As the variance of an abnormal VAG signal is commonly larger than that of a normal signal, the number of signal turns detected from the abnormal signal is smaller, which cannot describe the oscillations in the abnormal signal. To overcome such a drawback, the lowpass Butterworth filter and the fixed threshold method were introduced in the present study. With the fixed threshold, more signal turns would be detected from the abnormal VAG signals, which revealed the degree of higher variability in amplitude resulting from different knee joint disorders. Compared with the adaptive threshold, the fixed threshold could help physicians establish a straightforward but effective discriminant criterion in knee joint VAG signal monitoring.

Regarding the signal classification, the results of the single LS-SVM/LOO are much better than the previous studies using the logistic regression analysis with AR coefficients as features (accuracy: 68.9%) [17]; or with the energy, energy spread, frequency, and frequency spread features derived from the Gabor MP method (accuracy: 68.9%, *A*_*z*_: 0.68) [9]. The improvement of classification accuracy was largely contributed by the Natom (*P* = 0.0002) and TCFT (*P* = 0.0013) features developed in the present study.

Although the accurate rate of the Bagging 8.99% was lower than the LS-SVM/LOO, the ensemble provided an *A*_*z*_ of 0.8483, which was much larger than that of the LS-SVM/LOO (*A*_*z*_: 0.7523), which implied that the Bagging performed better on the prediction of the true positive (abnormal) signals associated with the knee joint disorders. On the other hand, it is worth noting that the Bagging ensemble did not outperform the five CSVMs. The Bagging ensemble was worse in accurate rate than either CSVM2 or CSVM5, and its *A*_*z*_ value was lower than that of CSVM1. The reason why the Bagging did not achieve the better performance may be related to the robustness of the LS-SVM. According to the remarks of Breiman's work [48], “Bagging stable classifiers is not a good idea”, because the Bagging ensemble with stable classifiers can only slightly improve the accuracy, but leading to more computational complexity.

The DWF-based ensemble produced higher classification accuracy and better ROC diagnostic performance than any of the CSVMs, along with the Bagging ensemble and the single LS-SVM/LOO. The ROC curve result of the DWF-based ensemble is consistently better than that of the RBFN classifier with the features of form factors, skewness, kurtosis, and entropy (*A*_*z*_: 0.8172) [11] or with the features of variance of mean-squared values and turns count with adaptive threshold (*A*_*z*_: 0.9174) [12] in the recent related studies. In addition, the DWF method is also comparable to the nonlinear strict 2-surface proximal classifier (*A*_*z*_: 0.95) proposed by Mu et al. [21]. The fusion weights in the DWF-based ensemble were dynamically optimized, which guaranteed the superiority of diagnostic performance.

## 7. Conclusion

 Analysis of VAG signals using advanced digital signal processing and pattern recognition techniques is able to provide distinct indicators of degenerative articular cartilage surfaces, and has high potential for noninvasive detection of knee joint pathology [53, 54]. In the feature extraction experiments, the features of Natom and TCFT, respectively, derived from the time-frequency wavelet MP decomposition and time-domain signal variability analysis are separable with significant *P* values. Using these features, the classification by means of the LS-SVM/LOO is superior to the logistic regression analysis used in the previous studies. In addition, we utilized the ensembles of classifiers to effectively ameliorate the overall classification performance. Compared with the most popular Bagging algorithm, the DWF-based ensemble used in the present study can significantly improve the classification accuracy and the ROC curve with higher *A*_*z*_ and lower SE values, over the entire VAG data set.

## Figures and Tables

**Table 1:** Classification results of different classifiers and ensemble systems on the testing data set. LS-SVM/LOO: the least-squares support vector machine evaluated with the leave-one-out (LOO) method. CSVM: the component least-squares support vector machines. DWF: the dynamic weighted fusion method. ROC curve: receiver operating characteristic curve.

Classifier	Accuracy (%)	Area under ROC curve (*A*_*z*_)	Standard error (SE)
LS-SVM/LOO	87.64	0.7523	0.0536
CSVM1	76.41	0.8578	0.0403
CSVM2	83.15	0.8772	0.0413
CSVM3	76.41	0.7884	0.0541
CSVM4	76.41	0.7064	0.0643
CSVM5	82.02	0.8844	0.0359
Bagging	78.65	0.8483	0.0474
DWF	88.76	0.9515	0.0244
